# Global Transcriptome and Co-Expression Network Analyses Revealed Hub Genes Controlling Seed Size/Weight and/or Oil Content in Peanut

**DOI:** 10.3390/plants12173144

**Published:** 2023-08-31

**Authors:** Lingli Yang, Li Yang, Yingbin Ding, Yuning Chen, Nian Liu, Xiaojing Zhou, Li Huang, Huaiyong Luo, Meili Xie, Boshou Liao, Huifang Jiang

**Affiliations:** The Key Laboratory of Biology and Genetic Improvement of Oil Crops, The Ministry of Agriculture and Rural Affairs, Oil Crops Research Institute of the Chinese Academy of Agricultural Sciences, Wuhan 430000, China; yanglingli1120@163.com (L.Y.); yangli2010119@163.com (L.Y.); dingyb1225@163.com (Y.D.); chenyuning@caas.cn (Y.C.); lnian0531@caas.cn (N.L.); zhouxiaojing@caas.cn (X.Z.); huangli01@caas.cn (L.H.); huaiyongluo@caas.cn (H.L.); xiemeili0101@163.com (M.X.); lboshou@hotmail.com (B.L.)

**Keywords:** peanut (*Arachis hypogaea* L.), seed development, transcriptome analysis, co-expression network, seed size/weight, oil content, hub genes

## Abstract

Cultivated peanut (*Arachis hypogaea* L.) is an important economic and oilseed crop worldwide, providing high-quality edible oil and high protein content. Seed size/weight and oil content are two important determinants of yield and quality in peanut breeding. To identify key regulators controlling these two traits, two peanut cultivars with contrasting phenotypes were compared to each other, one having a larger seed size and higher oil content (Zhonghua16, ZH16 for short), while the second cultivar had smaller-sized seeds and lower oil content (Zhonghua6, ZH6). Whole transcriptome analyses were performed on these two cultivars at four stages of seed development. The results showed that ~40% of the expressed genes were stage-specific in each cultivar during seed development, especially at the early stage of development. In addition, we identified a total of 5356 differentially expressed genes (DEGs) between ZH16 and ZH6 across four development stages. Weighted gene co-expression network analysis (WGCNA) based on DEGs revealed multiple hub genes with potential roles in seed size/weight and/or oil content. These hub genes were mainly involved in transcription factors (TFs), phytohormones, the ubiquitin–proteasome pathway, and fatty acid synthesis. Overall, the candidate genes and co-expression networks detected in this study could be a valuable resource for genetic breeding to improve seed yield and quality traits in peanut.

## 1. Introduction

Cultivated peanut (*Arachis hypogaea* L.) is an important grain legume that provides high-quality edible oil, rich protein, and other nutrients. In legumes, seed development is precisely modulated by both maternal and zygote signals, which, in coordination, regulate the growth of the embryo, endosperm, and seed coat [1]. The diploid embryo, triploid endosperm, and maternal integument grow in concert to control the seed size [2]. Seed size/weight is a crucial factor determining peanut yield and thus one of the major agronomic traits in peanut domestication and modern breeding. In addition, owing to their well-balanced fatty acid composition, peanuts are considered a functional food rich in specific antioxidants and mono- and polyunsaturated fatty acids [3]. Although the oil content of currently cultivated peanut varieties is usually high, there are still significant differences among different germplasms, ranging from 31.7 to 57.0% [4]. Hence, developing novel peanut cultivars with higher oil content is a prime target under the condition of limited planting area.

A large number of genes that regulate seed size/weight and/or oil content have been identified in *Arabidopsis thaliana* (Arabidopsis), *Glycine max* (soybean), *Oryza sativa* (rice), and *Zea mays* (maize), including genes related to transcription factors (TFs) and phytohormones [5,6,7,8], as well as genes involved in the ubiquitin–proteasome pathway and fatty acid biosynthesis [9,10,11]. For example, *GmAP2-1* and *GmAP2-4*, belonging to the AP2/ERF family, were identified as positively regulating seed weight and size in soybean [7]. The overexpression of *GmMYB73* was found to increase the oil content of *Arabidopsis* seeds by 5.9–17.9% and significantly enhance the 1000-seed weight [5]. Activation of the expression of the *Big Grain1* (*BG1*) gene, which regulates auxin transport, resulted in a significant increase in rice seed size [6]. Furthermore, the ubiquitin receptor protein encoding gene *DA1*, involved in the ubiquitin–proteasome pathway, is an inhibitor of the regulation of *Arabidopsis* seed size by restricting the cell proliferation cycle [9]. Suppressed expression of fatty acid dehydrogenase 2 (*FAD2*) and fatty acid elongase 1 (*FAE1*) involved in the de novo biosynthesis of fatty acids led to a slight decrease in the content of rapeseed oil and affected the accumulation of storage products [10]. However, few genes that influence seed size and/or oil content have been found in peanuts, which greatly restricts the breeding of high-quality peanut varieties.

In previous studies, genetic mapping was used to identify genomic loci controlling important traits in peanut, leading to the identification of multiple QTLs associated with yield [12,13,14,15] and oil content [16,17]. Nevertheless, it is challenging to detect candidate genes in QTLs that determine seed size/weight and/or oil content due to the complexity of peanut genome structure and the low-level polymorphism of molecular markers across different tetraploid *A. hypogaea* cultivars [12,18,19,20,21]. Owing to its reduced cost and high throughput nature, RNA-seq has been widely used to investigate transcriptome profiles in many plant species to assist in the identification of key genes for traits of interest [22,23,24,25,26,27]. For instance, comparative transcriptome analyses of wild and cultivated soybean varieties at the early and middle stages of seed maturation resulted in the identification of two genes potentially important for seed trait formation, *GA20OX* and *NFYA* [23]. A similar transcriptome comparison was conducted between large-seed and small-seed soybean cultivars at seed formation, growth, and early maturation stages, revealing several candidate genes that influence seed size, including TFs and phytohormones [24].

In addition, comparative transcriptome studies on peanuts have also been reported. For example, the transcriptome dynamics at 11 consecutive developmental stages of peanut pods showed that multiple genes involved in various pathways, such as gravitropism, photomorphogenesis, and response to stimuli, were differentially expressed along developmental gradients [22]. The transcriptome differences of two peanut varieties with distinct sucrose contents were explored at seven seed developmental stages, identifying 28 DEGs involved in sucrose metabolism [28]. Hub genes positively correlated with *Aspergillus flavus* resistance were identified in two peanut lines with contrasting genotypes via comparative transcriptome analysis [27]. Likewise, several candidate genes, including those encoding transcription factor TGA7, IAA-amino acid hydrolase, and pentatricopeptide repeat protein, were detected by a transcriptome study on two cultivated peanut accessions and their wild relative *Arachis monticola* at four stages of seed development [26]. However, studies to understand the molecular mechanisms underlying seed size and oil content in peanut through comprehensive transcriptome analysis are still largely unavailable.

In this study, comparative transcriptome analysis was conducted on two peanut cultivars with significant differences in seed size/weight and oil content at different stages of seed development. Transcriptome dynamics and gene co-expression networks associated with seed development were systematically investigated. Candidate genes potentially controlling seed size/weight and/or oil content were pinpointed. Overall, this study provides new insights into the molecular mechanisms underlying peanut seed development and forms a valuable resource for the genetic improvement of seed size/weight and oil content in peanut breeding.

## 2. Materials and Methods

### 2.1. Plant Materials and Sampling

Two peanut cultivars (*Arachis hypogaea* L.) with contrasting phenotypes in seed size/weight and oil content (namely, ZH16 (larger seed and higher oil content and ZH6 (smaller seed and lower oil content)) were grown in 2017 in an experimental field of the Oil Crops Research Institute, Wuhan, China. In order to mitigate the effects caused by environmental factors, we maintained consistent field management practices throughout the entire growth period. Daytime temperatures range from 25 °C to 35 °C, and the night temperature is not lower than 20 °C. According to a previous study (Pattee et al., 1974), seeds were collected with three biological replicates at 10, 20, 30, and 40 days after flowering (DAF), corresponding to the S1, S2, S3, and S4 stages, respectively. The seeds were very small and difficult to isolate at the S1 stage, while the entire seed remained flattened and white, or only one end turned pink, at the S2 stage. The seeds in the S3 stage were torpedo-shaped and the end of the kernel embryonic axis was generally pink, while the seeds in the final S4 stage were round-shaped and completely light pink. We sampled at least 30 seeds for each biological replicate at the S1 and S2 stages and 10 seeds for each biological replicate at the S3 and S4 stages. The harvested seeds were immediately frozen in liquid nitrogen and then stored at −80 °C until RNA isolation.

### 2.2. RNA-Seq Library Construction, Illumina Sequencing, and Differential Gene Expression Analysis

Total RNA was extracted using the TGuide plant RNA extraction kit (TIANGEN) following the manufacturer’s instructions. RNA purity and concentration were determined using a Nanodrop 2000c spectrophotometer and a Qubit 2.0 Flurometer. All 24 libraries (8 samples, 3 biological replicates) were sequenced on the Illumina HiSeq2000 platform. Raw reads in fastq format were firstly filtered using the NGS QC Toolkit v2.3 [29]. Obtained clean reads were then mapped to the cultivated peanut Tifrunner genome (https://data.legumeinfo.org/Arachis/hypogaea/ (accessed on 16 June 2023) [20]) by Hisat2 v2.0.5 [30]. Gene expression of known and novel genes was quantified as Fragments Per Kilobase of exon model per million mapped reads (FPKM) using StringTie v1.3.4 [31]. Principle component analysis (PCA) and correlation analysis were conducted with the princomp function and corrplot package in R v3.6.2, respectively. Genes whose expression differences reached the thresholds |log_2_(FoldChange)| > 1 and padj < 0.05 were defined as differentially expressed genes (DEGs) by DESeq2 [32]. Raw data of RNA-seq are available in the NCRI SRA database (PRJNA893583).

### 2.3. Identification of Stage-Specific Expression Genes

Stage-specific genes of two cultivars were identified using a stage specificity (SS) scoring algorithm, which compared the gene expression at one stage with the maximum expression at the other stages of seed development [25,33,34]. The SS scores ranged from 0 to 1. The higher the SS score of a gene at a certain stage, the more specific the expression of the gene at that stage. Genes with an SS score ≥ 0.5 were defined as being specifically expressed at a given developmental stage in each cultivar.

### 2.4. Functional Annotation and Enrichment Analysis 

The functional annotation file of the reference genome was downloaded from the website (https://data.legumeinfo.org/Arachis/hypogaea/ (accessed on 16 June 2023)). GO enrichment analysis was performed using BiNGO plugins in Cytoscape [35], and GO terms with *p* < 0.05 were considered to be significantly enriched.

### 2.5. Weighted Gene Co-Expression Network Analysis (WGCNA)

To explore the regulatory relationships across genes, WGCNA was performed on DEGs using R v3.6.2 [36]. Pairwise co-expressed genes with weighted values < 0.2 were removed via in-house perl scripts, and the resulting significant co-expression networks were finally visualized in Cytoscape v3.8.2 [37]. Modules were defined as gene clusters with high correlation coefficients among genes. Hub genes in each module were identified with the absolute value of kME (eigengene connectivity) greater than 0.8 (|kME| > 0.8).

## 3. Results

### 3.1. Phenotype Analysis of Two Peanut Cultivars ZH16 and ZH6 with Contrasting Seed Size/Weight and Oil Content

**Figure 1:**
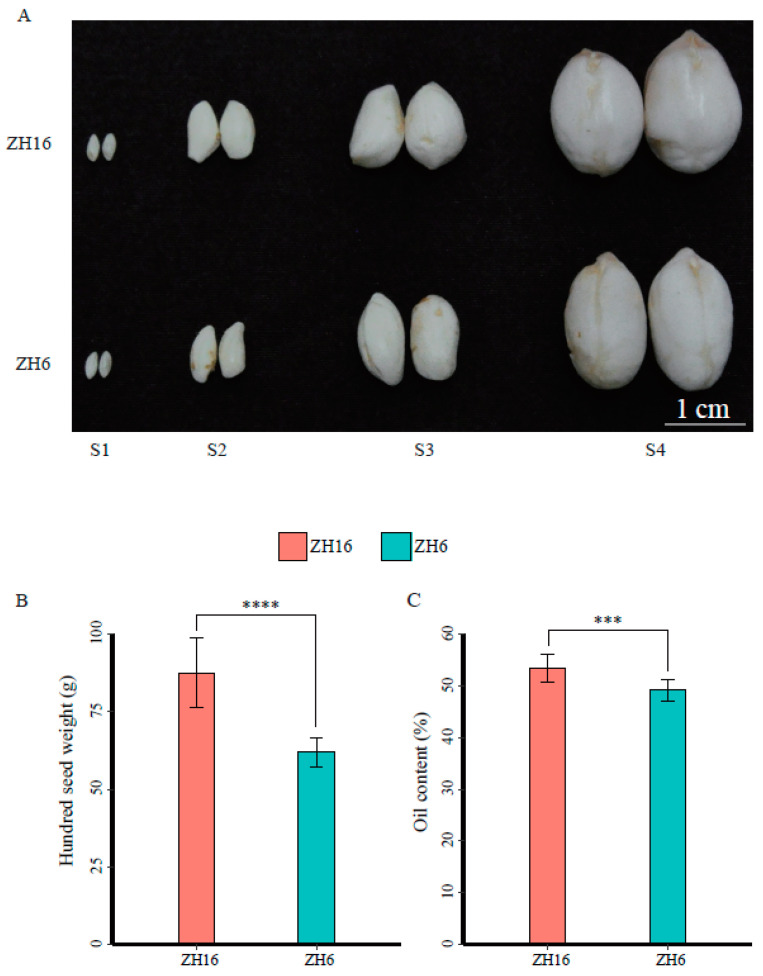
Phenotype differences between ZH16 and ZH6 at four stages of seed development. (**A**) Graphical display of seeds at different stages of development (S1–S4) between ZH16 and ZH6. (**B**) Average 100-seed weights (g) between ZH16 and ZH6. (**C**) Average oil content between ZH16 and ZH6. ***, *p* < 0.001; ****, *p* < 0.0001 (Student’s *t*-tests).

In this study, fresh seeds from two cultivars ZH16 and ZH6 at the S1, S2, S3, and S4 stages were collected to evaluate their seed size, while mature seeds were harvested to measure the hundred-seed weight and oil content. The results showed no significant difference in seed size between ZH16 and ZH6 at the S1 stage. With the extension of growth stages, the seed size of ZH16 was larger than that of ZH6 at the S2 stage, and this difference was more obvious at the S3 and S4 stages (Figure 1A). This phenomenon is in accordance with the significant difference in the hundred-seed weight between ZH16 and ZH6, which were 87.58 g and 61.92 g, respectively (Figure 1B). Moreover, a slight significant variation in terms of oil content was also found between these two accessions, with 53.44% and 49.13% in ZH16 and ZH6, respectively (Figure 1C).

### 3.2. Comparative Transcriptome Analysis of Seeds of ZH16 and ZH6 at Different Developmental Stages

To explore transcriptional dynamics of ZH16 and ZH6 during seed development, RNA-seq analysis was performed on seeds at the S1, S2, S3, and S4 stages of seed development. In total, about 810 million raw reads were generated, with an average of ~30 million reads per sample (Supplementary Table S1). After filtering low-quality reads, clean reads were aligned to the reference genome of cultivated peanut (Bertioli et al., 2019).

**Figure 2:**
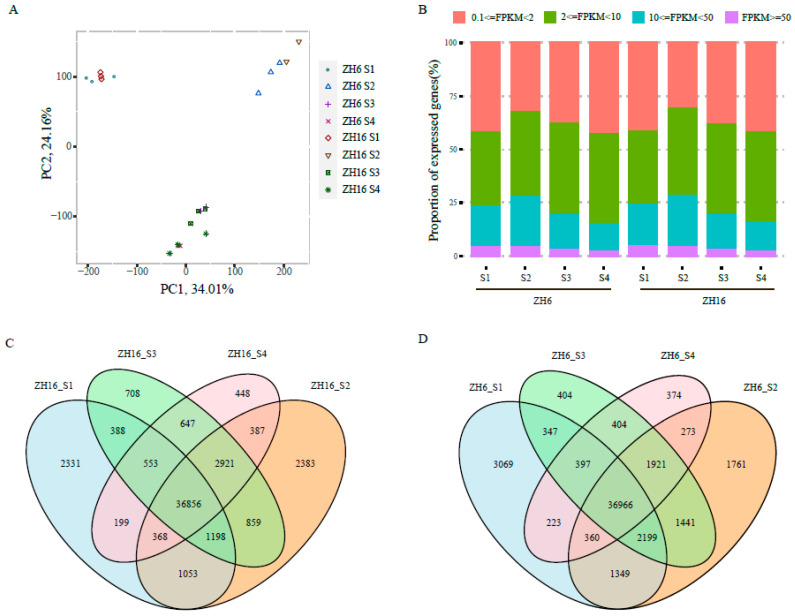
Global gene expression profiles in ZH16 and ZH6. (**A**) PCA plot showing clustering of gene transcript levels at four stages of seed development in ZH16 and ZH6. (**B**) Proportion of genes with different expression levels (based on FPKM). (**C**,**D**) Venn diagrams of expressed genes amongfour stages of seed development in cultivars ZH16 (**C**) and ZH6 (**D**).

To offer a comprehensive overview of the transcriptome dataset and to evaluate noise effects, we performed a principal component analysis (PCA) based on the average FPKM values of all expressed genes (Figure 2A). Samples of ZH16 and ZH6 at the same developmental stage were clustered together, indicating that the overall transcriptome dynamics of these two cultivars were similar at the same stage of seed development. Moreover, all samples from the S3 and S4 stages of both cultivars were grouped together, suggesting higher similarity in their transcriptome profiles. Correlation analysis based on FPKM showed high correlation coefficients across three different replicates of nearly all samples, except for ZH16 S2, ZH6 S3, and ZH6 S4, which only had high correlation between two biological replicates (Supplementary Figure S1A). Collectively, these results showed that our RNA-seq data were reliable and reproducible, and that they could be used for subsequent in-depth analyses.

A total of 53,434 genes were identified as expressed genes with FPKM values higher than 0.1 in at least one of the eight samples (Supplementary Table S2). Among them, 42,945, 46,025, 44,129, and 42,378 genes were found to be expressed in the ZH16 cultivar at the S1, S2, S3, and S4 stages of seed development, respectively (Supplementary Figure S1B). Similarly, 44 909, 46 270, 44 078, and 40 917 expressed genes were found to be expressed in the ZH6 cultivar at the S1, S2, S3, and S4 stages of seed development, respectively (Supplementary Figure S1B). Moreover, the proportion of genes with different expression levels was also similar between ZH16 and ZH6 at the same stage. Approximately 40% of the genes exhibited a low expression level (0.1 ≤ FPKM ≤ 2) in different developmental stages of the two cultivars (Figure 2B). With the seed development of each cultivar, the ratio of highly expressed genes (FPKM ≥ 10) dropped slightly (e.g., from 23% at S1 to 15% at the S4 stage), while the ratio of genes with moderate expression (2 ≤ FPKM < 10) increased marginally (e.g., from ~35% at S1 to ~42% at the S4 stage) (Figure 2B). The overlap and specificity of expressed genes in the four stages of ZH16 and ZH6 are displayed in Figure 2C,D. These results suggested large variations in genome-wide gene expression at different stages of seed development, which may be related to the observed differences in seed size/weight and oil content between ZH16 and ZH6.

### 3.3. Identification of Stage-Specific Expressed Genes during Seed Developmental Stages in ZH16 and ZH6

**Figure 3:**
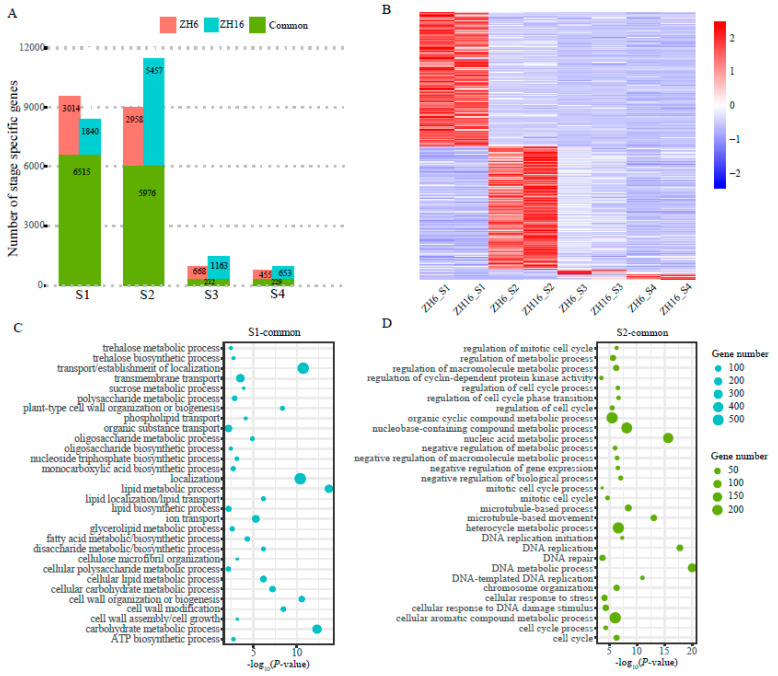
Genes with stage-specific expression during seed development in peanut cultivars ZH16 and ZH6. (**A**) Bar graph showing numbers of stage-specific expressed genes specifically or commonly present in ZH16 and/or ZH6 at each stage of seed development. (**B**) Heatmap showing the expression of common stage-specific expressed genes at different stages in ZH16 and ZH6. Color scale represents Z-score. (**C**,**D**) Enriched functional GO terms (biological process) of common stage-specific expressed genes in two cultivars at the S1 (**C**) and S2 (**D**) stages.

To explore the different transcriptional characteristics of the two cultivars at each stage of seed development, a stage specificity (SS) algorithm was used to identify stage-specific genes with an SS score ≥ 0.5. A total of 22,045 and 20,027 specifically expressed genes were identified in ZH16 and ZH6 at all stages of seed development, respectively (Supplementary Table S3). The numbers of stage-specific genes were notably different across the four different stages, varying from 882 (S4) to 11,433 (S2) for ZH16 and from 684 (S4) to 9529 (S1) for ZH6 (Figure 3A). Accordingly, the common stage-specific expressed genes in both cultivars ranged from 212 (S3) to 6515 (S1) (Figure 3A). The expression heatmap of all common stage-specific genes showed that the majority of these genes were highly expressed at the early developmental stages (S1, S2) in both cultivars (Figure 3B), which was consistent with a previous study on peanut [28]. Interestingly, more stage-specific genes were detected in ZH16 than in ZH6 at all stages except S1, with the largest increase at the S2 stage. These results suggest that each cultivar had its own independent developmental process, especially at the early stage.

To explore the specific function at different developmental stages, GO enrichment analysis was performed on common stage-specific genes in each stage of ZH16 and ZH6 (Figure 3C,D, Supplementary Figure S2). At the S1 stage, significant GO terms (*p* < 0.05) of common stage-specific genes were related to cell wall organization or biogenesis, cell wall modification, the carbohydrate metabolic process, the lipid metabolic process, and transport (Figure 3C). At the S2 stage, common stage-specific genes were mainly involved in cell cycle and division-related process, various metabolic processes, and the regulation of these processes (Figure 3D). Furthermore, for the S3 and S4 stages, the top GO terms included a variety of response and regulation processes, such as the response to hormone/chemical stimulus, the regulation of photomorphogenesis, and the hormone-mediated signaling pathway (Supplementary Figure S2).

### 3.4. Identification of Differentially Expressed Genes during Seed Development in Two Peanut Cultivars

**Figure 4:**
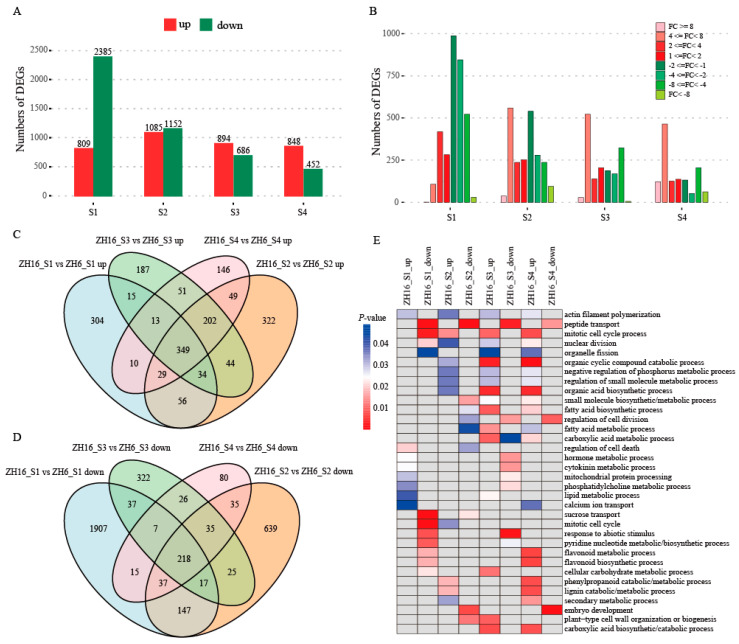
DEGs between ZH16 and ZH6 at different seed developmental stages. (**A**) Number of up-regulated and down-regulated genes. (**B**) Distribution of Log_2_FC values of DEGs. (**C**,**D**) Venn diagrams showing numbers of DEGs concurrently or specifically expressed among four stages. (**E**) Enriched GO terms (biological process) of up- and down-regulated genes. The color scale represents significance (corrected *p*-value).

To investigate transcriptional differences between ZH16 and ZH6 cultivars, we performed pairwise comparisons of the two cultivars at four seed developmental stages. Compared with ZH6, a total of 5222 differentially expressed genes (DEGs) were identified in ZH16 across four stages, among which 1810 genes were up-regulated and 3546 genes were down-regulated (Supplementary Table S4). The number of DEGs was highest at the S1 stage (3194), followed by the S2 stage (2237), S3 stage (1580), and S4 stage (1300) (Figure 4A). Notably, the number of up-regulated DEGs was greater than that of down-regulated DEGs at the S3 and S4 stages, whereas the opposite was found at stage S1 (Figure 4A). DEGs grouped in accordance with their log_2_FoldChange (FC) were shown to be unevenly distributed across all stages. FC values of the most up-regulated genes were between two- and four-fold or between four- and eight-fold, while the absolute FC values of down-regulated genes were mostly between one- and two-fold or between four- and eight-fold (Figure 4B). Of all these DEGs, only 349 up-regulated and 218 down-regulated genes were overlapping across all four stages (Figure 4C,D). These results revealed the distinct patterns of gene expression between ZH16 and ZH6 at different stages of seed development.

GO enrichment analysis of DEGs showed that several biological processes were commonly or uniquely enriched at different seed developmental stages (Figure 4E, Supplementary Figure S3). We observed that in all developmental stages except S1, up-regulated DEGs were mainly enriched in various functional terms related to cell division, such as the mitotic cell cycle process, nuclear division, and organic cyclic compound catabolic process. Various primary and secondary metabolic/biosynthetic processes, including carboxylic acid, fatty acid, lipid, flavonoid, phenylpropanoid, and lignin, were significantly enriched for highly expressed genes at the S3 and/or S4 stages. However, genes involved in hormone/cytokinin/phosphatidylcholine metabolic processes were up-regulated at the S1 stage and down-regulated at the S3 stage.

### 3.5. Co-Expression Network Analysis of DEGs by WGCNA

**Figure 5:**
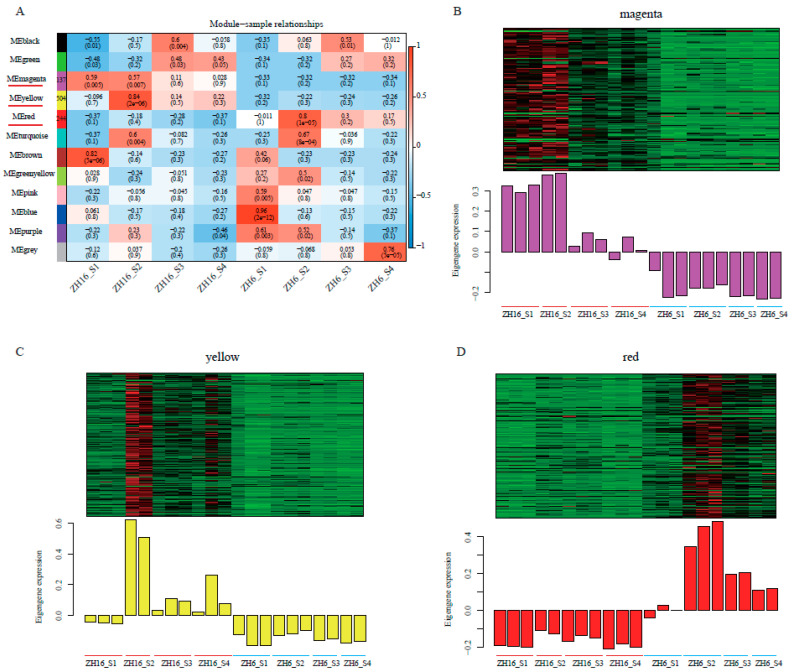
WGCNA of DEGs between ZH16 and ZH6 at each seed developmental stage. (**A**) Module–sample relationships. The number of genes within key modules is indicated next to the module name. Color bars represent negative (blue) and positive (red) correlations. (**B**–**D**) Expression patterns of DEGs in magenta (**B**), yellow (**C**), and red (**D**) modules.

To further explore potential key genes that determine differences in seed size/weight and/or oil content between two cultivars, WGCNA was performed based on the expression levels of 4367 DEGs at four developmental stages after filtering unknown genes. Based on the soft threshold power β = 9, a scale-free network was constructed, resulting in the generation of 11 co-expression modules (Figure 5, Supplementary Figure S4). The modules were color-coded, and the grey module contained genes not assigned to any other modules. The gene numbers of different modules varied greatly, ranging from 84 to 1176. Correlation analysis of module–sample relationships revealed that the magenta module was positively associated with larger-seed and higher-oil ZH16 at the S1 and S2 stages (*r* = 0.59, *p* = 0.005; *r* = 0.57, *p* = 0.007) and the yellow module was positively associated with ZH16 at the S2 stage (*r* = 0.84, *p* = 2 × 10^−6^), while the red module was strongly positively correlated with smaller-seed and lower-oil ZH6 at the S2 stage (*r* = 0.8, *p* = 1 × 10^−5^) (Figure 5A).

Subsequently, the expression patterns of genes within these three modules at different stages of seed development in ZH16 and ZH6 were visualized in heatmaps (Figure 5B–D). During seed development, especially at the S2 stage, the gene expression of the magenta and yellow modules was higher in larger-seed and higher-oil ZH16 than in ZH6, indicating their positive effect in seed size/weight and oil content (Figure 5B,C). However, the expression of genes in the red module was higher in all stages of smaller-seed and lower-oil ZH6, especially in the S2 stage (Figure 5D), suggesting their negative roles in these two traits. Overall, the genes within the magenta, yellow, and red modules may be closely related to the difference in size/weight and/or oil content between ZH16 and ZH6 cultivars, so further analysis of the genes in these three modules is of high significance. GO enrichment analysis was performed on the genes belonging to these three key modules to elucidate their gene function. Magenta and yellow modules positively correlated with ZH16 were significantly enriched in functional terms of cell cycle, mRNA modification, fatty acid metabolic process, and ubiquitin-dependent proteolysis process. For the genes with lower expression within the red module in ZH16, the top terms were enriched in ribosome biogenesis and rRNA metabolic process.

### 3.6. Identifying Hub Genes Associated with Seed Size/Weight and/or Oil Content within Key Modules

**Figure 6:**
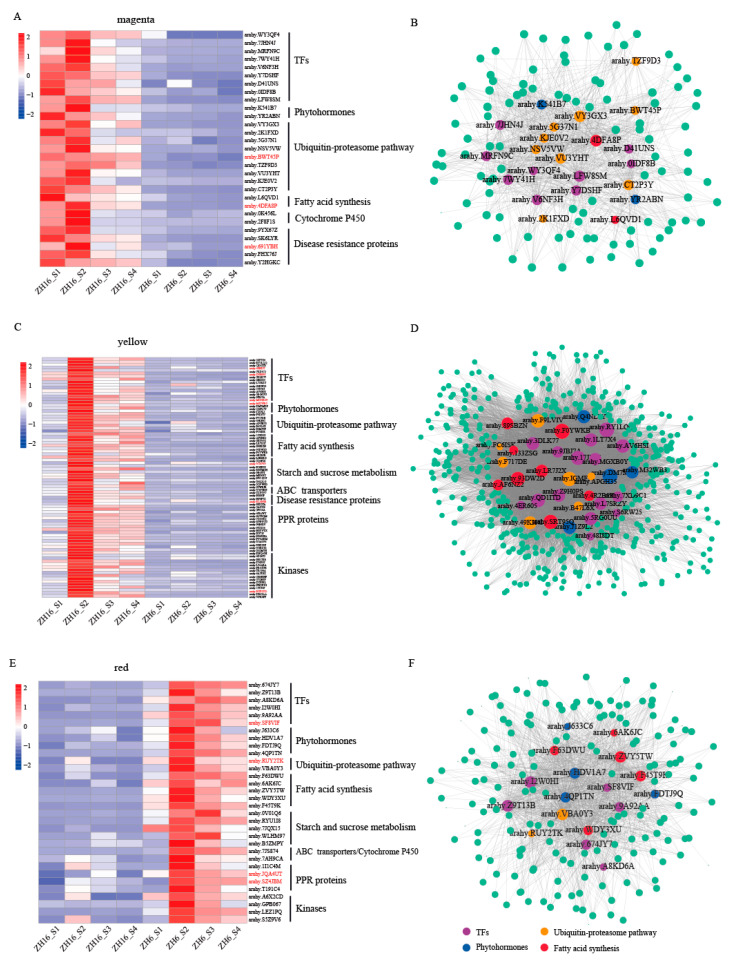
Heatmap and co-expression networks of DEGs within three key modules. (**A**,**C**,**E**) Heatmaps of hub genes. Genes overlapping with reported QTLs are marked in red. (**B**,**D**,**F**) Co-expression networks. Purple, blue, orange, and red nodes represent hub genes involved in TFs, phytohormones, the ubiquitin–proteasome pathway, and fatty acid synthesis, respectively. Node size represents connectivity.

To identify key candidates related to size/weight and oil content in magenta, yellow, and red modules, hub genes were identified based on their high kME values (|kME| > 0.8). According to functional annotations and reported genes associated with these two traits in other species (Supplementary Table S5), hub genes involved in TFs, phytohormones, the ubiquitin–proteasome pathway, and fatty acid synthesis may influence size/weight and/or oil content (Figure 6A–F; Supplementary Table S6). In addition, we collected known QTLs or candidate genes associated with seed size/weight and/or oil content reported in previous studies (Supplementary Table S7). A total of 14 hub genes involved in the aforementioned pathways were found to overlap with these loci or candidate genes, as shown in red in Figure 6.

#### 3.6.1. TFs

A total of twenty-four TFs in the magenta and yellow modules and six TFs in the red module were identified as hub genes belonging to the WRKY, AP2, bHLH, MADS-box, MYB, ARF, SBP, and SNF2 families (Figure 6A–F; Supplementary Table S6). Among them, two WRKY family genes (*arahy.Y7DSHF* and *arahy.V6NF3H*) showed significantly higher expression in ZH16 than in ZH6. The *Arabidopsis wrky10* mutant exhibited smaller seeds due to early cellularization of the endosperm, which inhibited the proliferation of embryonic cells [38]. Two genes (*arahy.WY3QF4* and *arahy.1LT7 ×* 4), encoding AP2-like ethylene-responsive TF, had notably higher expression in ZH16; they were previously shown to play a positive role in regulating seed weight and size in soybean [7,39]. The overexpression of AP2/EREBP TFs *BnWRI1* and *GmWRI1a* led to enhanced seed oil content in rapeseed and soybean, respectively [40,41]. Furthermore, the transcript abundances of three MADS-box TFs (*arahy.L7SRZY*, *arahy.S6RW25*, and *arahy.7JHN4J*) were remarkably higher in ZH16. These TFs were reported to play a role in seed size via modulating endosperm cellularization in rice [42]. Two MYB genes (*arahy. MRFN9C* and *arahy. 17JY83*) and one ARF (*arahy.RY1LQQ*) with a higher expression level in ZH16 were previously shown to determine seed size/weight in plants [43,44].

#### 3.6.2. Phytohormones and the Ubiquitin–Proteasome Pathway

Several hub genes related to phytohormones, such as auxin, gibberellin, and cytokinin, were identified in this study (Figure 6A–F; Supplementary Table S6) and were recently reported to regulate seed size [45,46,47]. Three hub genes (*arahy.RY1LQQ*, *arahy.HDV1A7*, and *arahy.FDTJ9Q*) involved in auxin response or transport were detected to potentially control the seed size in maize and rice [6,8]. Three histidine kinase 1 encoding genes (*arahy.Q4NL7Y*, *arahy.DM7MDF*, and *arahy.K541B7*), receptors of cytokinin in *Arabidopsis* (Riefler et al. 2006), showed significantly higher expression in ZH16. A gibberellin 20 oxidase 2 encoding gene (*arahy.M32WB3*) was found to exhibit strikingly higher transcript level in ZH16. It should be noted that *OsGA3ox2* was remarkably up-regulated in wild-type rice than in *sng1* mutant with reduced grain weight [47].

Furthermore, a number of hub genes involved in the ubiquitin–proteasome pathway were found (Figure 6A–F; Supplementary Table S6). For example, two genes (*arahy.VY3GX3* and *arahy.2K1FXD*) encoding E3 ubiquitin–protein ligases showed significantly higher expression in ZH16, which was recently found to influence seed size through the starch synthesis pathway in wheat [48]. Zinc finger (C3HC4-type RING finger) family protein encoding genes *arahy.B47L6X* and *arahy.APGH35*, functioning as E3 ubiquitin ligases, had higher transcript abundance in ZH16. Five F-box proteins encoding hub genes derived from the magenta and yellow modules showed a higher expression level in ZH16 with larger-seed and higher-oil content. These genes were characterized to have substrate recognition specificity in ubiquitin-mediated proteolysis [49].

#### 3.6.3. Fatty acid Synthesis

A number of DEGs involved in fatty acid biosynthesis were identified, such as those encoding fatty acid hydroxylase, fatty acid desaturase, 3-ketoacyl-CoA synthase, long chain acyl-CoA synthetase, and acyl-CoA thioesterase (Figure 6A–F; Supplementary Table S6). Two genes (*arahy.6AK6JC* and *arahy.ZVY5TW*) encoding acyl-CoA thioesterase were found to have lower expression level in ZH16 than in ZH6, while fatty acid desaturase encoding gene *arahy.93DW2D* showed the opposite. These two enzymes were previously demonstrated to influence rapeseed oil content [10,50]. In addition, DEGs involved in starch and sucrose metabolism were also detected, including those encoding beta glucosidase, glycoside hydrolase, fructose-bisphosphate aldolase, and xyloglucan endotransglucosylase. Sucrose is used to produce dihydroxyacetone phosphate and acetyl-CoA as raw materials for TAG synthesis, which is a key step in the formation of seed oil [51].

## 4. Discussion

In this study, we aimed to determine the molecular mechanisms underlying seed size/weight and oil content by performing comparative transcriptome analysis on two peanut cultivars with contrasting phenotypes at four stages of seed development. A total of 5222 DEGs were identified between ZH16 and ZH6 across the four stages of seed development analyzed. Combined with co-expression networks analysis, hub genes in three key modules were identified, which were mainly involved in TFs, phytohormones, the ubiquitin–proteasome pathway, fatty acid synthesis, cytochrome P450 proteins, ABC transporters, PPR proteins, and receptor-like kinases.

### 4.1. Roles of Important Genes in Determining Seed Size/Weight

#### 4.1.1. TFs

TFs play a key role in regulating plant life cycle activities and adaptation to the environment, such as controlling plant seed development [52,53,54]. In this study, a total of 30 TFs were identified as both DEGs and hub genes within several co-expression modules (Figure 6A–F; Supplementary Table S6). Among them, two genes (*arahy.Y7DSHF* and *arahy.V6NF3H*) belonging to WRKY family showed significantly higher expression in ZH16 than in ZH6. *GmWRKY15a* and *OsWRKY36* were found to regulate seed size in soybean and rice, respectively [52,55]. Another two bHLH TFs (*arahy.7XD9C1* and *arahy.48I8DT*) were also identified to have higher transcript levels in ZH16. Functionally deficient mutant of the bHLH TF *RGE1* (*ZOU*) resulted in slow embryo growth, eventually leading to the production of small and wrinkled seeds in *Arabidopsis* and maize [56,57]. Another bHLH TF *TaPGS1* was also reported to positively regulate grain weight and size in rice and wheat [58]. These results indicate that TFs play an important role in the regulation of plant seed size/weight.

#### 4.1.2. Phytohormones

Many phytohormones, such as auxin, gibberellin, cytokinin, and brassinolide, have been reported to regulate plant seed growth and development [46,59,60,61]. In our study, six DEGs related to auxin or cytokinin were identified, including those encoding auxin response factor, auxin transport protein, and histidine kinase (Figure 6A–F; Supplementary Table S6). *ZmARF12* is an inhibitor of cell division during seed development, and its defective mutant could lead to increased seed size/weight in maize [8], whereas the *arf25* mutant resulted in decreased grain weight and size in wheat [62]. The transport of auxin to maternal tissue is a key driver of seed coat development, thus affecting seed size in *Arabidopsis* [63]. Moreover, cytokinins may influence seed size by regulating the growth of embryonic cells during seed development [64,65]. A gene (*BnaA03G37960D*) encoding cytokinin receptor histidine kinase in rapeseed was the candidate gene for 1000-seed weight by linkage and association analyses [45]. These results illuminate that phytohormones are crucial for plant seed development and the regulation of seed size/weight.

#### 4.1.3. The Ubiquitin–Proteasome Pathway

It has been reported that the ubiquitin–proteasome pathway is involved in the improvement of crop yield by regulating seed agronomic traits, such as seed size/weight [66]. Some DEGs associated with the ubiquitin–proteasome pathway were found in this study (Figure 6A–F; Supplementary Table S6). The *Arabidopsis* ubiquitin receptor protein encoding gene *DA1* was an inhibitor that regulated seed size by limiting cell proliferation [9]. Similarly, *DA2* encodes an ubiquitin ligase that negatively regulated seed size in *Arabidopsis* and cotton [11,67]. *TaGW2-6A*, encoding an E3 ubiquitin ligase, has been reported to interact with *TaAGPS* in wheat to influence seed size through the starch synthesis pathway [48].

#### 4.1.4. Other Important Genes

We also found that two genes (*arahy.2F8F1S* and *arahy.0K456L*) encoding cytochrome P450 proteins had higher expression levels in ZH16. Previously, *KLU* (*CYP78A5*) and *EOD3* (*CYP78A6*) encoding cytochrome P450 monooxygenases were found to positively regulate seed size in *Arabidopsis* [68,69]. Overexpression of *GmCYP78A72*/*GmCYP78A5* and *TaCYP78A3* could increase seed size in soybean and wheat, respectively [24,70,71]. Three genes (*arahy.S5WRJR*, *arahy.VYP3BN*, and *arahy.TZU5V7*) related to the ABC transport pathway displayed relatively higher expression levels in ZH16. An ABCC3 transporter gene *CaABCC3 (6)* could affect seed size by regulating the transport of glutathione conjugates in chickpea seed cells [72]. Additionally, we also found other DEGs, including those encoding PPR proteins and receptor-like kinases. It was previously reported that PPR protein encoding genes *Dek39* and *Emp18*, involved in RNA editing, were essential for seed development in maize [73,74]. PPR proteins may also be related to the increase in seed size/weight of cultivated peanut [26]. The receptor-like kinase ERECTA (ER) and LecRK-VIII.2 have been shown to positively regulate seed size by modulating the proliferation of outer integument cells, and they were upstream regulators of MAPK signaling pathway in *Arabidopsis* [75,76].

### 4.2. Roles of Key Genes Related to Fatty Acid Biosynthesis in Determinating Seed Oil Content

Oil synthesis can be divided into three stages during seed development. Firstly, acetyl-CoA is used as the initial substrate to synthesize long-chain fatty acids in plastids. Subsequently, long-chain fatty acids are transported to the endoplasmic reticulum (ER) to assemble triacylglycerol (TAG). Finally, triacylglycerol combines with oil body proteins to form oil bodies [77]. In this study, we identified a number of DEGs involved in fatty acid biosynthesis, including those encoding enzymes and TFs (Figure 6A–F; Supplementary Table S6). It has been reported that the knockout of *KASIII* (β-ketoacyl-ACP synthase) and *FATB* (acyl-ACP thioesterase) in rapeseed resulted in an increase in contents of medium chain fatty acids [50]. Reduced expression of *FAD2* (fatty acid dehydrogenase 2) and *FAE1* (fatty acid elongase 1) in rapeseed using RNAi caused a slight decrease in seed oil content, an increase in oleic acid content, and a decrease in erucic acid content [10]. Similarly, low expression of fatty acid desaturase and elongase in crambe could largely increase seed oleic acid content [78].

AP2-like ethylene-responsive TF encoding genes *arahy.WY3QF4* and *arahy.1LT7×4* were found to show significantly higher expression in ZH16 in our study. The AP2/EREBP TF *ZmWRI1* was regulated by upstream regulators *LEC2* and *LEC1* and positively regulated the downstream target genes *PI-PKβ1*, *BCCP2*, *ACP1,* and *KAS1*, which were mainly involved in glycolysis and fatty acid synthesis in maize [79]. Overexpression of *BnWRI1* and *GmWRI1a* led to enhanced seed oil content in rapeseed and soybean via complex co-expression gene networks related to fatty acid biosynthesis, respectively [40,41,80]. Interestingly, a similar phenomenon was found in our work, where *arahy.1LT7×4* and several genes involved in fatty acid biosynthesis were shown to co-express within the yellow module. The results suggest that AP2-like ethylene-responsive TF may influence seed oil content by regulating downstream target genes involved in fatty acid biosynthesis in plants.

### 4.3. Genes Affecting Both Seed Oil Content and Seed Size/Weight

In our work, several DEGs were found to potentially contribute to both seed oil content and seed size/weight, including AP2/EREBP and MYB TFs. AP2 family members have been reported to regulate ovule development and fatty acid synthesis in seed, thereby determining seed size/weight [7,81]. Specific overexpression of the AP2/EREBP TF (*BnWRI1*) in *B. napus* led to enhanced oil content and seed size/weight in matured seeds [40]. Additionally, overexpression of *GmMYB73* could increase seed oil content and seed size/weight in transgenic *Arabidopsis* [5]. These results suggest that AP2/EREBP and MYB TFs may not only increase seed oil content but also seed size/weight.

## 5. Conclusions

In this study, we performed comparative transcriptome analysis on two peanut cultivars with contrasting seed size/weight and oil content to identify the key candidate genes determining these two important traits. Our results revealed that a number of crucial genes, including those encoding TFs, phytohormones, ubiquitin-mediated proteolysis related proteins, cytochrome P450 proteins, and ABC transporters, play a crucial role in regulating seed size/weight. Several genes involved in fatty acid biosynthesis were detected to contribute to seed oil biosynthesis. Additionally, some genes may both affect oil content and seed size, such as AP2/EREBP and MYB TFs. Taken together, this study provides comprehensive information underlying seed size/weight and oil content and potentially serves as a beneficial resource for genetic breeding to develop peanut cultivars with enhanced yield and quality.

## Data Availability

Sequencing data are deposited in the Sequence Read Archive of NCBI with the BioProject accession number PRJNA893583. The original contributions presented in this study are included in the article/Supplementary Material; further inquiries can be directed to the corresponding author/s.

## Supplementary Materials

The following supporting information can be downloaded at: https://www.mdpi.com/article/10.3390/plants12173144/s1, Supplementary Figure S1. Correlation statistics and number of expressed genes at different stages of seed development between ZH16 and ZH6. (A) Spearman correlation coefficient (SCC) of RNA-seq data. (B) Bar plot showing number of expressed genes. Supplementary Figure S2. Enriched GO terms (biological process) of common stage-specific expressed genes in two cultivars at the S3 and S4 stages. The x-axis represents -log10 (*p*-value). Bubble size represents the number of enriched genes. Supplementary Figure S3. Specifically enriched GO terms of up-regulated (A) or down-regulated (B) genes at each stage of seed development. Supplementary Figure S4. Network topology analysis. (A) Sample clustering. (B) Topology and connectivity based on soft thresholding powers. (C) Hierarchical clustering of co-expression modules. Each leaf on the tree represents a gene. The major branches form 11 color-coded modules. (D) Correlation heatmap across different modules. Supplementary Table S1. Summary of RNA-seq data. Supplementary Table S2. The lists of genes expressed (FPKM > 0.1) in at least one of the eight samples. Supplementary Table S3. Information regarding stage-specific expressed genes in ZH16 and ZH6. Supplementary Table S4. Information regarding DEGs between ZH16 and ZH6. Supplementary Table S5. Information regarding reported genes related to seed size/weight and/or oil content in other species. Supplementary Table S6. Information regarding hub genes within three key modules. Supplementary Table S7. Information regarding reported QTL or genes related to seed size/weight and/or oil content in peanut.

## References

[1] Li N., Xu R., Li Y. (2019). Molecular Networks of Seed Size Control in Plants. Annu. Rev. Plant Biol..

[2] Bleckmann A., Alter S., Dresselhaus T. (2014). The beginning of a seed: Regulatory mechanisms of double fertilization. Front. Plant Sci..

[3] Akhtar S., Khalid N., Ahmed I., Shahzad A., Suleria H.A. (2014). Physicochemical characteristics, functional properties, and nutritional benefits of peanut oil: A review. Crit. Rev. Food Sci. Nutr..

[4] Yol E., Ustun R., Golukcu M., Uzun B. (2017). Oil Content, Oil Yield and Fatty Acid Profile of Groundnut Germplasm in Mediterranean Climates. J. Am. Oil Chem. Soc..

[5] Liu Y.F., Li Q.T., Lu X., Song Q.X., Lam S.M., Zhang W.K., Ma B., Lin Q., Man W.Q., Du W.G. (2014). Soybean GmMYB73 promotes lipid accumulation in transgenic plants. BMC Plant Biol..

[6] Liu L., Tong H., Xiao Y., Che R., Xu F., Hu B., Liang C., Chu J., Li J., Chu C. (2015). Activation of Big Grain1 significantly improves grain size by regulating auxin transport in rice. Proc. Natl. Acad. Sci. USA.

[7] Jiang W.B., Zhang X.J., Song X.W., Yang J.F., Pang Y.Z. (2020). Genome-Wide Identification and Characterization of APETALA2/Ethylene-Responsive Element Binding Factor Superfamily Genes in Soybean Seed Development. Front. Plant Sci..

[8] Wang Y.Z., Nie L.H., Ma J., Zhou B., Han X.H., Cheng J.L., Lu X.M., Fan Z.F., Li Y.L., Cao Y.Y. (2022). Transcriptomic Variations and Network Hubs Controlling Seed Size and Weight During Maize Seed Development. Front. Plant Sci..

[9] Li Y., Zheng L., Corke F., Smith C., Bevan M.W. (2008). Control of final seed and organ size by the DA1 gene family in Arabidopsis thaliana. Genes Dev..

[10] Shi J.H., Lang C.X., Wang F.L., Wu X.L., Liu R.H., Zheng T., Zhang D.Q., Chen J.Q., Wu G.T. (2017). Depressed expression of FAE1 and FAD2 genes modifies fatty acid profiles and storage compounds accumulation in *Brassica napus* seeds. Plant Sci..

[11] Huang L., Yang S.X., Wu L.Y., Xin Y., Song J.K., Wang L., Pei W.F., Wu M., Yu J.W., Ma X.Y. (2022). Genome-Wide Analysis of the GW2-Like Genes in Gossypium and Functional Characterization of the Seed Size Effect of GhGW2-2D. Front. Plant Sci..

[12] Luo H.Y., Guo J.B., Ren X.P., Chen W.G., Huang L., Zhou X.J., Chen Y.N., Liu N., Xiong F., Lei Y. (2018). Chromosomes A07 and A05 associated with stable and major QTLs for pod weight and size in cultivated peanut (*Arachis hypogaea* L.). Theor. Appl. Genet..

[13] Zhang S., Hu X., Miao H., Chu Y., Cui F., Yang W., Wang C., Shen Y., Xu T., Zhao L. (2019). QTL identification for seed weight and size based on a high-density SLAF-seq genetic map in peanut (*Arachis hypogaea* L.). BMC Plant Biol..

[14] Gangurde S.S., Wang H., Yaduru S., Pandey M.K., Fountain J.C., Chu Y., Isleib T., Holbrook C.C., Xavier A., Culbreath A.K. (2020). Nested-association mapping (NAM)-based genetic dissection uncovers candidate genes for seed and pod weights in peanut (*Arachis hypogaea*). Plant Biotechnol. J..

[15] Jadhav M.P., Gangurde S.S., Hake A.A., Yadawad A., Mahadevaiah S.S., Pattanashetti S.K., Gowda M.V.C., Shirasawa K., Varshney R.K., Pandey M.K. (2021). Genotyping-by-Sequencing Based Genetic Mapping Identified Major and Consistent Genomic Regions for Productivity and Quality Traits in Peanut. Front. Plant Sci..

[16] Shasidhar Y., Vishwakarma M.K., Pandey M.K., Janila P., Variath M.T., Manohar S.S., Nigam S.N., Guo B.Z., Varshney R.K. (2017). Molecular Mapping of Oil Content and Fatty Acids Using Dense Genetic Maps in Groundnut (*Arachis hypogaea* L.). Front. Plant Sci..

[17] Liu N., Guo J.B., Zhou X.J., Wu B., Huang L., Luo H.Y., Chen Y.N., Chen W.G., Lei Y., Huang Y. (2020). High-resolution mapping of a major and consensus quantitative trait locus for oil content to a ~ 0.8-Mb region on chromosome A08 in peanut (*Arachis hypogaea* L.). Theor. Appl. Genet..

[18] Bertioli D.J., Cannon S.B., Froenicke L., Huang G.D., Farmer A.D., Cannon E.K.S., Liu X., Gao D.Y., Clevenger J., Dash S. (2016). The genome sequences of Arachis duranensis and Arachis ipaensis, the diploid ancestors of cultivated peanut. Nat. Genet..

[19] Luo H.Y., Xu Z.J., Li Z.D., Li X.P., Lv J.W., Ren X.P., Huang L., Zhou X.J., Chen Y.N., Yu J.Y. (2017). Development of SSR markers and identification of major quantitative trait loci controlling shelling percentage in cultivated peanut (*Arachis hypogaea* L.). Theor. Appl. Genet..

[20] Bertioli D.J., Jenkins J., Clevenger J., Dudchenko O., Gao D., Seijo G., Leal-Bertioli S.C.M., Ren L., Farmer A.D., Pandey M.K. (2019). The genome sequence of segmental allotetraploid peanut *Arachis hypogaea*. Nat. Genet..

[21] Zhuang W.J., Chen H., Yang M., Wang J.P., Pandey M.K., Zhang C., Chang W.C., Zhang L.S., Zhang X.T., Tang R.H. (2019). The genome of cultivated peanut provides insight into legume karyotypes, polyploid evolution and crop domestication. Nat. Genet..

[22] Chen X.P., Yang Q.L., Li H.F., Li H.Y., Hong Y.B., Pan L.J., Chen N., Zhu F.H., Chi X.Y., Zhu W. (2016). Transcriptome-wide sequencing provides insights into geocarpy in peanut (*Arachis hypogaea* L.). Plant Biotechnol. J..

[23] Lu X., Li Q.T., Xiong Q., Li W., Bi Y.D., Lai Y.C., Liu X.L., Man W.Q., Zhang W.K., Ma B. (2016). The transcriptomic signature of developing soybean seeds reveals the genetic basis of seed trait adaptation during domestication. Plant J..

[24] Du J., Wang S.D., He C.M., Zhou B., Ruan Y.L., Shou H.X. (2017). Identification of regulatory networks and hub genes controlling soybean seed set and size using RNA sequencing analysis. J. Exp. Bot..

[25] Garg R., Singh V.K., Rajkumar M.S., Kumar V., Jain M. (2017). Global transcriptome and coexpression network analyses reveal cultivar-specific molecular signatures associated with seed development and seed size/weight determination in chickpea. Plant J..

[26] Li Z.F., Zhang X.G., Zhao K.K., Zhao K., Qu C.X., Gao G.Q., Gong F.P., Ma X.L., Yin D.M. (2021). Comprehensive Transcriptome Analyses Reveal Candidate Genes for Variation in Seed Size/Weight During Peanut (*Arachis hypogaea* L.) Domestication. Front. Plant Sci..

[27] Cui M.J., Han S.Y., Wang D., Haider M.S., Guo J.J., Zhao Q., Du P., Sun Z.Q., Qi F.Y., Zheng Z. (2022). Gene Co-expression Network Analysis of the Comparative Transcriptome Identifies Hub Genes Associated with Resistance to Aspergillus flavus L. in Cultivated Peanut (*Arachis hypogaea* L.). Front. Plant Sci..

[28] Li W.T., Huang L., Liu N.A., Pandey M.K., Chen Y.N., Cheng L.Q., Guo J.B., Yu B.L., Luo H.Y., Zhou X.J. (2021). Key Regulators of Sucrose Metabolism Identified through Comprehensive Comparative Transcriptome Analysis in Peanuts. Int. J. Mol. Sci..

[29] Patel R.K., Jain M. (2012). NGS QC Toolkit: A Toolkit for Quality Control of Next Generation Sequencing Data. PLoS ONE.

[30] Kim D., Paggi J.M., Park C., Bennett C., Salzberg S.L. (2019). Graph-based genome alignment and genotyping with HISAT2 and HISAT-genotype. Nat. Biotechnol..

[31] Pertea M., Pertea G.M., Antonescu C.M., Chang T.C., Mendell J.T., Salzberg S.L. (2015). StringTie enables improved reconstruction of a transcriptome from RNA-seq reads. Nat. Biotechnol..

[32] Love M.I., Huber W., Anders S. (2014). Moderated estimation of fold change and dispersion for RNA-seq data with DESeq2. Genome Biol..

[33] Ma C., Xin M.M., Feldmann K.A., Wang X.F. (2014). Machine Learning-Based Differential Network Analysis: A Study of Stress-Responsive Transcriptomes in Arabidopsis. Plant Cell.

[34] Zhan J.P., Thakare D., Ma C., Lloyd A., Nixon N.M., Arakaki A.M., Burnett W.J., Logan K.O., Wang D.F., Wang X.F. (2015). RNA Sequencing of Laser-Capture Microdissected Compartments of the Maize Kernel Identifies Regulatory Modules Associated with Endosperm Cell Differentiation. Plant Cell.

[35] Maere S., Heymans K., Kuiper M. (2005). BiNGO: A Cytoscape plugin to assess overrepresentation of Gene Ontology categories in Biological Networks. Bioinformatics.

[36] Langfelder P., Horvath S. (2008). WGCNA: An R package for weighted correlation network analysis. BMC Bioinform..

[37] Kohl M., Wiese S., Warscheid B. (2011). Cytoscape: Software for Visualization and Analysis of Biological Networks. Data Min. Proteom. Stand. Appl..

[38] Luo M., Dennis E.S., Berger F., Peacock W.J., Chaudhury A. (2005). MINISEED3 (MINI3), a WRKY family gene, and HAIKU2 (IKU2), a leucine-rich repeat (LRR) KINASE gene, are regulators of seed size in Arabidopsis. Proc. Natl. Acad. Sci. USA.

[39] Assefa T., Otyama P.I., Brown A.V., Kalberer S.R., Kulkarni R.S., Cannon S.B. (2019). Genome-wide associations and epistatic interactions for internode number, plant height, seed weight and seed yield in soybean. BMC Genom..

[40] Wu X.L., Liu Z.H., Hu Z.H., Huang R.Z. (2014). BnWRI1 coordinates fatty acid biosynthesis and photosynthesis pathways during oil accumulation in rapeseed. J. Integr. Plant Biol..

[41] Wang Z.K., Wang Y.Z., Shang P., Yang C., Yang M.M., Huang J.X., Ren B.Z., Zuo Z.H., Zhang Q.Y., Li W.B. (2022). Overexpression of Soybean GmWRI1a Stably Increases the Seed Oil Content in Soybean. Int. J. Mol. Sci..

[42] Chen C., Begcy K., Liu K., Folsom J.J., Wang Z., Zhang C., Walia H. (2016). Heat stress yields a unique MADS box transcription factor in determining seed size and thermal sensitivity. Plant Physiol..

[43] Gupta M., Bhaskar P.B., Sriram S., Wang P.H. (2017). Integration of omics approaches to understand oil/protein content during seed development in oilseed crops. Plant Cell Rep..

[44] Khemka N., Rajkumar M.S., Garg R., Jain M. (2021). Genome-wide profiling of miRNAs during seed development reveals their functional relevance in seed size/weight determination in chickpea. Plant Direct.

[45] Wang H., Yan M., Xiong M., Wang P.F., Liu Y., Xin Q., Wan L.L., Yang G.S., Hong D.F. (2020). Genetic dissection of thousand-seed weight and fine mapping of cqSW.A03-2 via linkage and association analysis in rapeseed (*Brassica napus* L.). Theor. Appl. Genet..

[46] Wang Y.Q., Zhang M.N., Du P., Liu H., Zhang Z.X., Xu J., Qin L., Huang B.Y., Zheng Z., Dong W.Z. (2022). Transcriptome analysis of pod mutant reveals plant hormones are important regulators in controlling pod size in peanut (*Arachis hypogaea* L.). Peerj.

[47] Yun P., Li Y.B., Wu B., Zhu Y., Wang K.Y., Li P.B., Gao G.J., Zhang Q.L., Li X.H., Li Z.F. (2022). OsHXK3 encodes a hexokinase-like protein that positively regulates grain size in rice. Theor. Appl. Genet..

[48] Lv Q., Li L.Q., Meng Y., Sun H.M., Chen L.P., Wang B.X., Li X.J. (2022). Wheat E3 ubiquitin ligase TaGW2-6A degrades TaAGPS to affect seed size. Plant Sci..

[49] Nguyen K.M., Busino L. (2020). The Biology of F-box Proteins: The SCF Family of E3 Ubiquitin Ligases. Adv. Exp. Med. Biol..

[50] Stoll C., Lühs W., Zarhloul M.K., Brummel M., Spener F., Friedt W. (2006). Knockout of KASIII regulation changes fatty acid composition in canola (*Brassica napus*). Eur. J. Lipid Sci. Technol..

[51] Apriyanto A., Compart J., Zimmermann V., Alseekh S., Fernie A.R., Fettke J. (2022). Indication that starch and sucrose are biomarkers for oil yield in oil palm (Elaeis guineensis Jacq.). Food Chem..

[52] Gu Y.Z., Li W., Jiang H.W., Wang Y., Gao H.H., Liu M., Chen Q.S., Lai Y.C., He C.Y. (2017). Differential expression of a WRKY gene between wild and cultivated soybeans correlates to seed size. J. Exp. Bot..

[53] Jo L., Pelletier J.M., Harada J.J. (2019). Central role of the LEAFY COTYLEDON1 transcription factor in seed development. J. Integr. Plant Biol..

[54] Jiang L.Z., Liu C.Y., Fan Y., Wu Q., Ye X.L., Li Q., Wan Y., Sun Y.X., Zou L., Xiang D.B. (2022). Dynamic transcriptome analysis suggests the key genes regulating seed development and filling in Tartary buckwheat (*Fagopyrum tataricum* Garetn.). Front. Genet..

[55] Lan J., Lin Q.B., Zhou C.L., Ren Y.K., Liu X., Miao R., Jing R.N., Mou C.L., Nguyen T., Zhu X.J. (2020). Small grain and semi-dwarf 3, a WRKY transcription factor, negatively regulates plant height and grain size by stabilizing SLR1 expression in rice. Plant Mol. Biol..

[56] Kondou Y., Nakazawa M., Kawashima M., Ichikawa T., Yoshizumi T., Suzuki K., Ishikawa A., Koshi T., Matsui R., Muto S. (2008). RETARDED GROWTH OF EMBRYO1, a new basic helix-loop-helix protein, expresses in endosperm to control embryo growth. Plant Physiol..

[57] Grimault A., Gendrot G., Chamot S., Widiez T., Rabille H., Gerentes M.F., Creff A., Thevenin J., Dubreucq B., Ingram G.C. (2015). ZmZHOUPI, an endosperm-specific basic helix-loop-helix transcription factor involved in maize seed development. Plant J. Cell Mol. Biol..

[58] Guo X.J., Fu Y.X., Lee Y.R.J., Chern M., Li M.L., Cheng M.P., Dong H.X., Yuan Z.W., Gui L.X., Yin J.J. (2022). The PGS1 basic helix-loop-helix protein regulates Fl3 to impact seed growth and grain yield in cereals. Plant Biotechnol. J..

[59] Zhang Y., Zhang Y.J., Yang B.J., Yu X.X., Wang D., Zu S.H., Xue H.W., Lin W.H. (2016). Functional characterization of GmBZL2 (AtBZR1 like gene) reveals the conserved BR signaling regulation in Glycine max. Sci. Rep..

[60] Cao J., Li G., Qu D., Li X., Wang Y. (2020). Into the Seed: Auxin Controls Seed Development and Grain Yield. Int. J. Mol. Sci..

[61] Wu Y., Sun Z.Q., Qi F.Y., Tian M.D., Wang J., Zhao R.F., Wang X., Wu X.H., Shi X.L., Liu H.F. (2022). Comparative transcriptomics analysis of developing peanut (*Arachis hypogaea* L.) pods reveals candidate genes affecting peanut seed size. Front. Plant Sci..

[62] Jia M.L., Li Y.A., Wang Z.Y., Tao S., Sun G.L., Kong X.C., Wang K., Ye X.G., Liu S.S., Geng S.F. (2021). TaIAA21 represses TaARF25-mediated expression of TaERFs required for grain size and weight development in wheat. Plant J..

[63] Figueiredo D.D., Batista R.A., Roszak P.J., Hennig L., Kohler C. (2016). Auxin production in the endosperm drives seed coat development in Arabidopsis. Elife.

[64] Werner T., Motyka V., Laucou V., Smets R., Van Onckelen H., Schmulling T. (2003). Cytokinin-deficient transgenic Arabidopsis plants show multiple developmental alterations indicating opposite functions of cytokinins in the regulation of shoot and root meristem activity. Plant Cell.

[65] Riefler M., Novak O., Strnad M., Schmulling T. (2006). Arabidopsis cytokinin receptor mutants reveal functions in shoot growth, leaf senescence, seed size, germination, root development, and cytokinin metabolism. Plant Cell.

[66] Varshney V., Majee M. (2022). Emerging roles of the ubiquitin-proteasome pathway in enhancing crop yield by optimizing seed agronomic traits. Plant Cell Rep..

[67] Disch S., Anastasiou E., Sharma V.K., Laux T., Fletcher J.C., Lenhard M. (2006). The E3 ubiquitin ligase BIG BROTHER controls arabidopsis organ size in a dosage-dependent manner. Curr. Biol. CB.

[68] Anastasiou E., Kenz S., Gerstung M., MacLean D., Timmer J., Fleck C., Lenhard M. (2007). Control of plant organ size by KLUH/CYP78A5-dependent intercellular signaling. Dev. Cell.

[69] Fang W., Wang Z., Cui R., Li J., Li Y. (2012). Maternal control of seed size by EOD3/CYP78A6 in Arabidopsis thaliana. Plant J. Cell Mol. Biol..

[70] Ma M., Wang Q., Li Z., Cheng H., Li Z., Liu X., Song W., Appels R., Zhao H. (2015). Expression of TaCYP78A3, a gene encoding cytochrome P450 CYP78A3 protein in wheat (*Triticum aestivum* L.), affects seed size. Plant J. Cell Mol. Biol..

[71] Zhao B., Dai A., Wei H., Yang S., Wang B., Jiang N., Feng X. (2016). Arabidopsis KLU homologue GmCYP78A72 regulates seed size in soybean. Plant Mol. Biol..

[72] Basu U., Upadhyaya H.D., Srivastava R., Daware A., Malik N., Sharma A., Bajaj D., Narnoliya L., Thakro V., Kujur A. (2019). ABC Transporter-Mediated Transport of Glutathione Conjugates Enhances Seed Yield and Quality in Chickpea. Plant Physiol..

[73] Li X.J., Gu W., Sun S.L., Chen Z.L., Chen J., Song W.B., Zhao H.M., Lai J.S. (2018). Defective Kernel 39 encodes a PPR protein required for seed development in maize. J. Integr. Plant Biol..

[74] Li X.L., Huang W.L., Yang H.H., Jiang R.C., Sun F., Wang H.C., Zhao J., Xu C.H., Tan B.C. (2019). EMP18 functions in mitochondrial atp6 and cox2 transcript editing and is essential to seed development in maize. New Phytol..

[75] Xiao W.J., Hu S., Zou X.X., Cai R.Q., Liao R., Lin X.X., Yao R.F., Guo X.H. (2021). Lectin receptor-like kinase LecRK-VIII.2 is a missing link in MAPK signaling-mediated yield control. Plant Physiol..

[76] Wu X.D., Cai X.B., Zhang B.W., Wu S.T., Wang R.J., Li N., Li Y.H., Sun Y., Tang W.Q. (2022). ERECTA regulates seed size independently of its intracellular domain via MAPK-DA1-UBP15 signaling. Plant Cell.

[77] Hills M.J. (2004). Control of storage-product synthesis in seeds. Curr. Opin. Plant Biol..

[78] Li X.Y., Mei D.S., Liu Q., Fan J., Singh S., Green A., Zhou X.R., Zhu L.H. (2016). Down-regulation of crambe fatty acid desaturase and elongase in Arabidopsis and crambe resulted in significantly increased oleic acid content in seed oil. Plant Biotechnol. J..

[79] Shen B., Allen W.B., Zheng P., Li C., Glassman K., Ranch J., Nubel D., Tarczynski M.C. (2010). Expression of ZmLEC1 and ZmWRI1 increases seed oil production in maize. Plant Physiol..

[80] Chen L., Zheng Y.H., Dong Z.M., Meng F.F., Sun X.M., Fan X.H., Zhang Y.F., Wang M.L., Wang S.M. (2018). Soybean (Glycine max) WRINKLED1 transcription factor, GmWRI1a, positively regulates seed oil accumulation. Mol. Genet. Genom..

[81] Ohto M.A., Floyd S.K., Fischer R.L., Goldberg R.B., Harada J.J. (2009). Effects of APETALA2 on embryo, endosperm, and seed coat development determine seed size in Arabidopsis. Sex. Plant Reprod..

