# Supplementary material for: Global Transcriptome and Co-Expression Network Analyses Revealed Hub Genes Controlling Seed Size/Weight and/or Oil Content in Peanut
Source: Plants (Basel). 2023 Aug 31;12(17):3144. doi: 10.3390/plants12173144 (PMC10490140; doi:10.3390/plants12173144)
Supplement: Supplementary file 1 [file plants-12-03144-s001.zip › plants-2482064 Supplementary Figures.pdf]

plants MDPI

Supplementary Materials

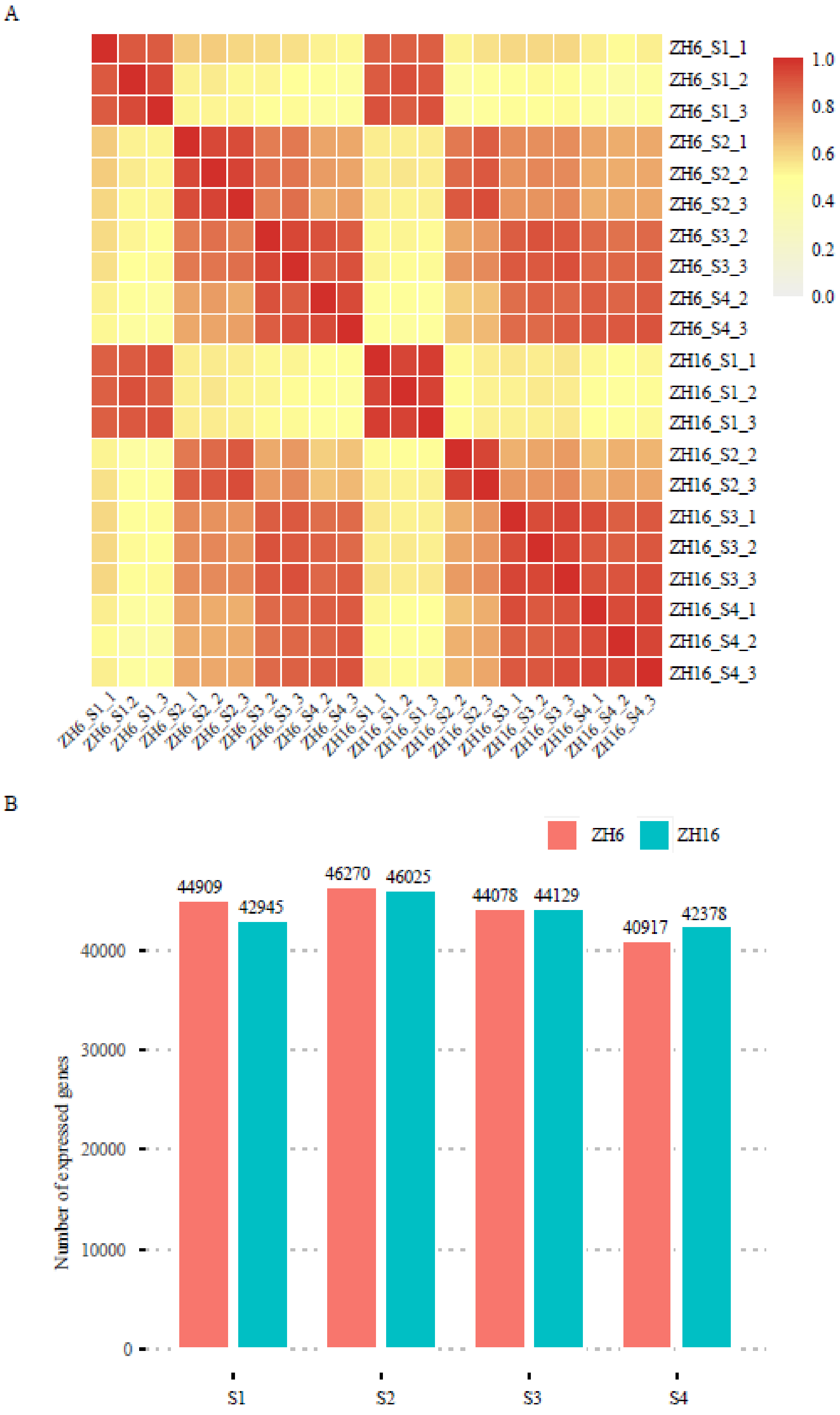

Figure S1. Correlation statistics and number of expressed genes at different stages of seed development between ZH16 and ZH6. (A) Spearman correlation coefficient (SCC) of RNA-seq data. (B) Bar plot showing number of expressed genes.

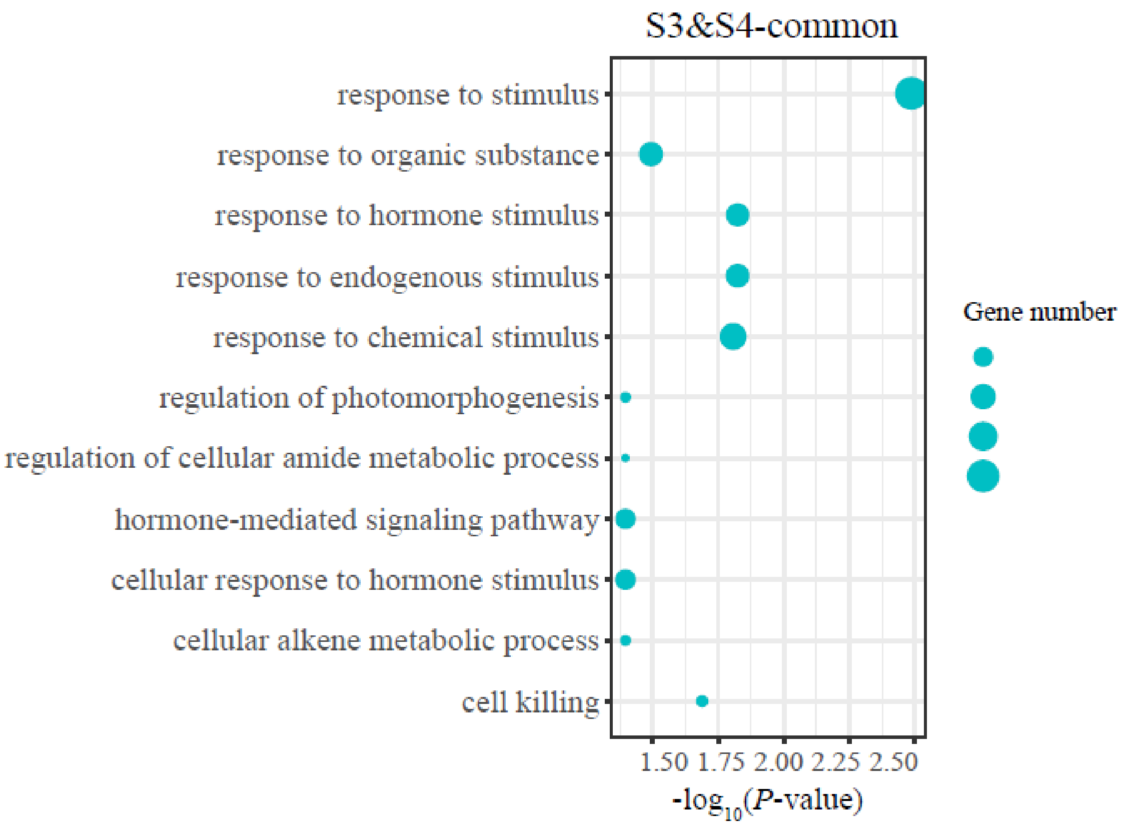

Figure S2. Enriched GO terms (biological process) of common stage-specific expressed genes in two cultivars at the S3 and S4 stages. The x-axis represents -log10 (*p*-value). Bubble size represents the number of enriched genes.

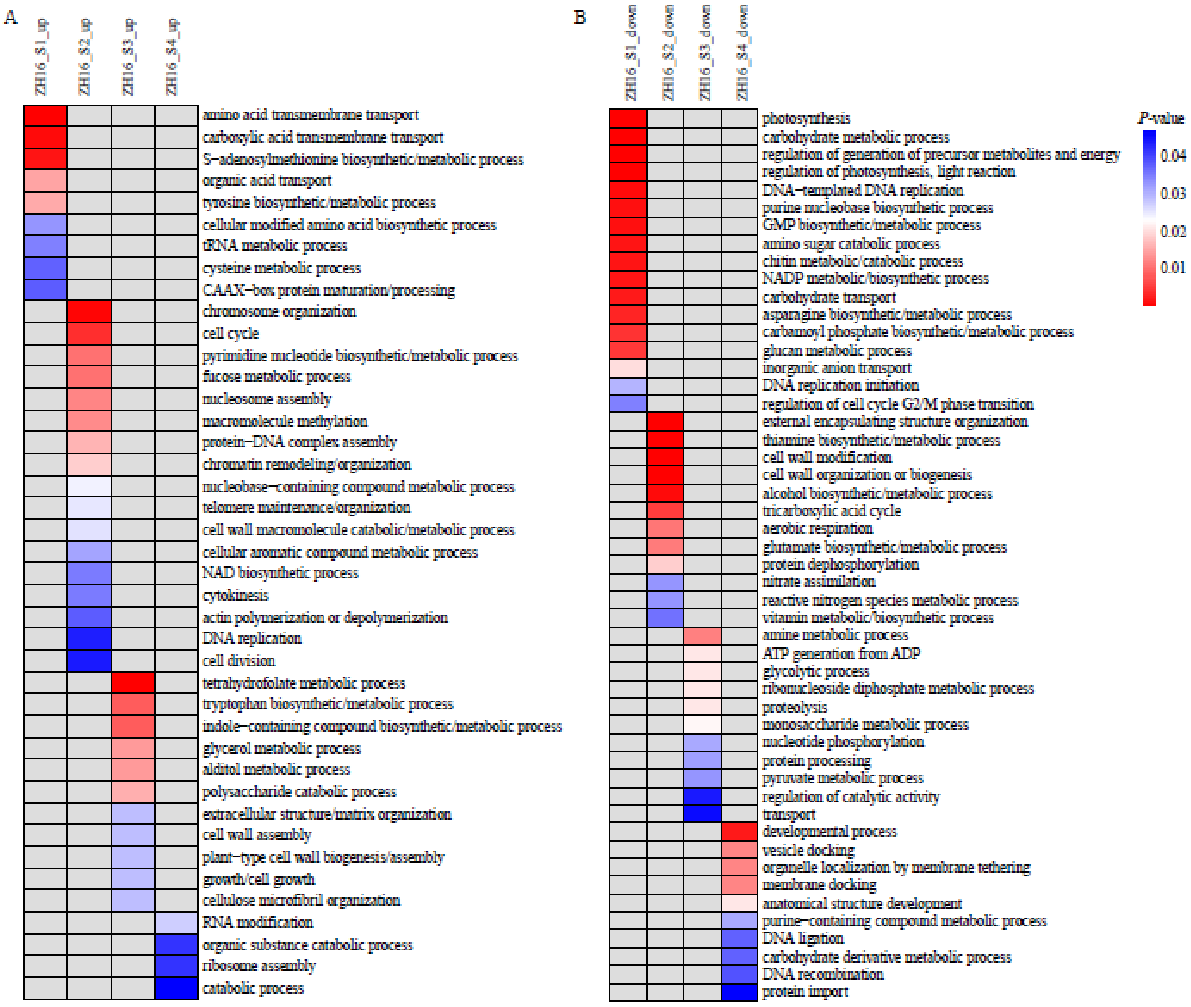

Figure S3. Specifically enriched GO terms of up-regulated (A) or down-regulated (B) genes at each stage of seed development.

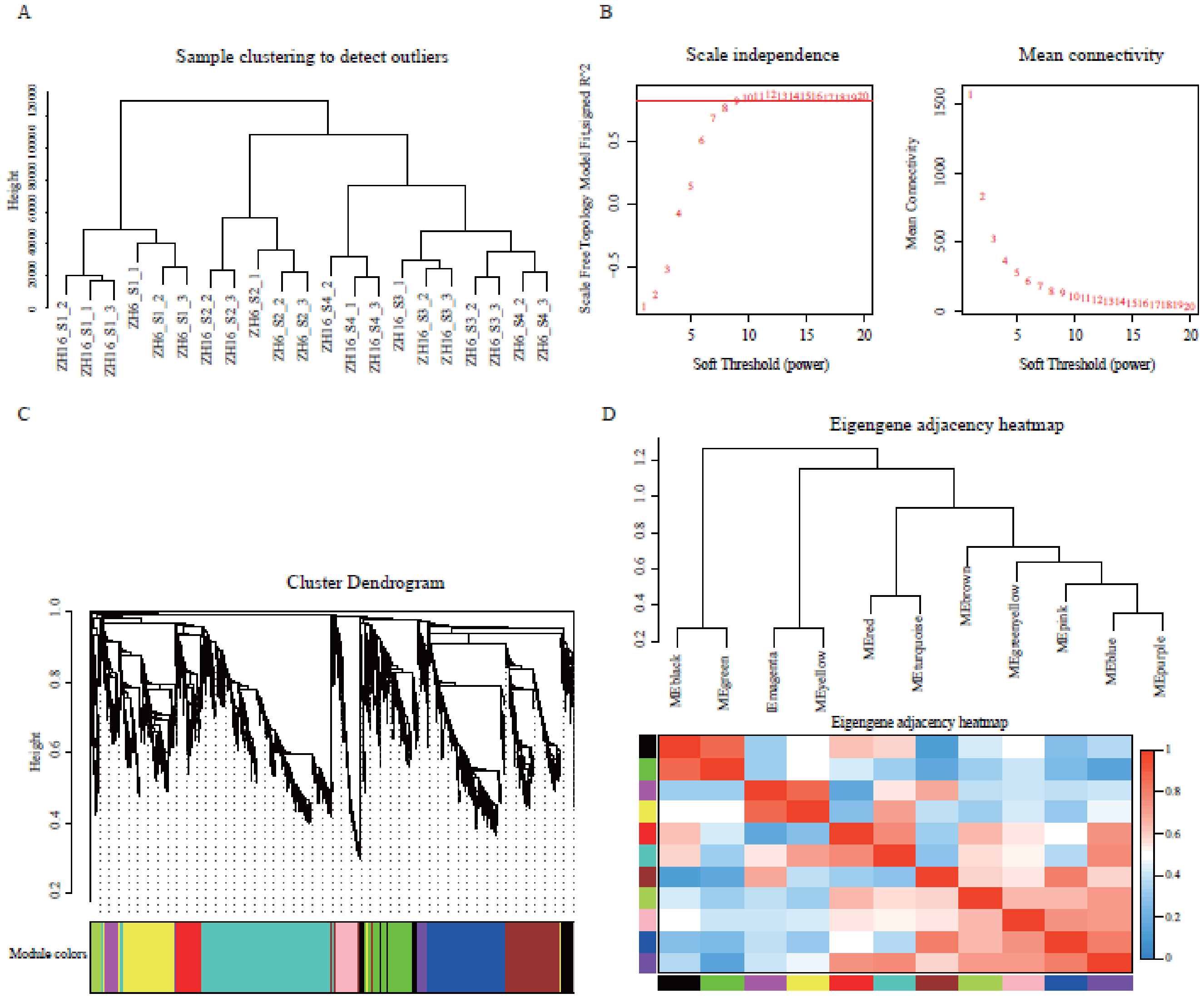

Figure S4. Network topology analysis. (A) Sample clustering. (B) Topology and connectivity based on soft thresholding powers. (C) Hierarchical clustering of co-expression modules. Each leaf on the tree represents a gene. The major branches form 11 color-coded modules. (D) Correlation heatmap across different modules.
